# Diffrential Diagnosis of Hepatic Hydrothorax by ^99m^Tc Sulfur Colloid Peritoneal Scintigraphy: Two Cases

**DOI:** 10.4021/gr2009.08.1310

**Published:** 2009-07-20

**Authors:** Cem Aygun, .Hakan Demir, Omer Senturk

**Affiliations:** a Department of Internal Medicine and Division of Gastroenterology, Kocaeli University Medical Faculty, Umuttepe/ Kocaeli, Turkey; b Department of Nuclear Medicine, Kocaeli University Medical Faculty, Umuttepe/ Kocaeli, Turkey

**Keywords:** Hepatic hydrothorax, ^99m^Tc sulfur colloid peritoneal scintigraphy

## Abstract

Large symptomatic pleural effusions can be seen in cirrhotic patients with ascites. In this report, two cirrhotic patients with ascites and large pleural effusions were evaluated. To determine the cause of pleural effusion and to understand whether there was a communication of fluid between peritoneal and pleural cavities, ^99m^Tc sulfur colloid peritoneal scintigraphy was used. According to the scintigraphic results, the diagnosis of hepatic hydrothorax and tuberculosis was confirmed easily and the treatments of the patients were managed rapidly.

## Introduction

A large effusion in a patient with cirrhotic ascites is described as hepatic hydrothorax which is a complication of cirrhosis and found in about 4% to 6% of cirrhotic patients in the absence of primary pulmonary, pleural, or cardiac disease [1-4]. Usually, it is located in the right hemithorax, but it can also be seen on the left side, bilaterally and even in the absence of ascites [5, 6is caused by direct passage of ascitic fluid from ]. It the abdominal cavity to the pleural space through transdiaphragmatic defects [7]. Diagnosis of hydrothorax is crucial since it could lead to hazardous complications such as bacterial pleuritis or empyema [8]. Patients with a long history of stable cirrhosis and a sudden development of pleural effusions should be suspected of hepatic hydrothorax as well as other causes that have precipitated fluid collection such as malignancy and tuberculosis.

Although the exact mechanism of hepatic hydrothorax is controversial, it is probably due to the ascites fluid that is transported directly into the pleural space. A number of different mechanisms have been proposed to explain the development of hepatic hydrothorax, including: hypoalbuminemia and a decrease in colloid osmotic pressure; leakage of plasma from hypertensive azygos veins; lymphatic leakage from thoracic duct; passage of fluid from peritoneal cavity to the pleural space via lymphatic channels in the diaphragm; and passage of fluid into the pleural space directly via defects in the diaphragm [1-7].

Among the different mechanisms proposed, it is mostly accepted that peritoneal fluid flows into the pleural space directly, along a pressure gradient through congenital or acquired fenestrations connecting these two spaces. The mechanism can be explained in such a way that the fenestrations in the tendinous portion of the diaphragm are covered with pleuroperitoneum and they are raised into blisters as intra abdominal pressure rises by ascites. Eventually blisters rupture and microscopic defects may appear. Positive intra abdominal pressure coupled with negative intrathoracic pressure causes a direct flow of fluid from abdominal cavity into the pleura. A pleural effusion develops if the flow exceeds the absorptive capacity of pleura [4].

In clinical practice, presence of communication between pleural and peritoneal cavities may be successfully demonstrated by peritoneal scintigraphy. In this technique, firstly, radiolabeled tracer should be simply introduced into the ascitic fluid by paracentesis. Later, scintigraphic images of abdomino-thoracic region are obtained by gamma camera. Using this technique, demonstration of the presence of a communication between abdomen and thoracic cavities can be quickly confirmed [9-11].

With this study we presented two cases of pleural effusion in which ^99m^Tc sulfur colloid peritoneal scintigraphy was used to detect whether there is such a passage of ascites fluid from the peritoneal cavity to the pleural space via the diaphragmatic defects.

## Patients

Two (one hepatitis B virus and one alcohol-related) cirrhotic patients with ascites and pleural effusions were evaluated to determine the cause of fluid accumulation and were searched whether there was a communication between peritoneal and pleural cavities. In addition to clinical evidence of ascites and pleural effusion, patients were also confirmed by paracentesis and thoracentesis of fluids. Protein content of ascitic and pleural fluids was used to estimate whether the fluid was due to chronic liver disease.

## Scintigraphic analysis

After aspiration of ascitic fluid, approximately 37 MBq of ^99m^Tc sulfur colloid instilled the peritoneal cavity under sterile conditions. Patients were then asked to turn frequently from side to side for proper mixing of radiopharmaceutical into the ascitic fluid. Approximately 10 - 15 min after injection of the tracer, each patient was positioned supine under a large field-of-view single-head gamma camera (Adac Argus Epic, ADAC Laboratories, Milpitas, CA, USA), fitted with a low-energy general purpose collimator. An anterior abdominal image was acquired in a 256 x 256 byte matrix for 400 kcounts to confirm the presence of tracer in the peritoneal cavity. Thereafter, images of upper abdomen and chest in anterior and posterior positions were obtained at 30 min intervals for 6 h. Twenty-four-hour images were also acquired in the second patient (Case 2) since no activity was noticed in the chest region until 6 h. Markers placed at the costal margins and xyphoid to show anatomic orientation. Scintigraphic images were evaluated by two experienced nuclear medicine physicians for the presence or absence of radiotracer in pleural regions.

## Case 1

**Figure 1:**
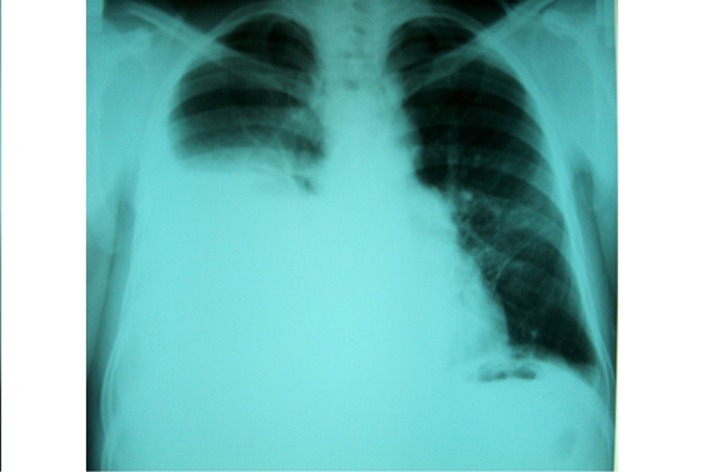
PA chest radiograph of the case 1: a large pleural effusion was seen in the right hemithorax.

**Figure 2:**
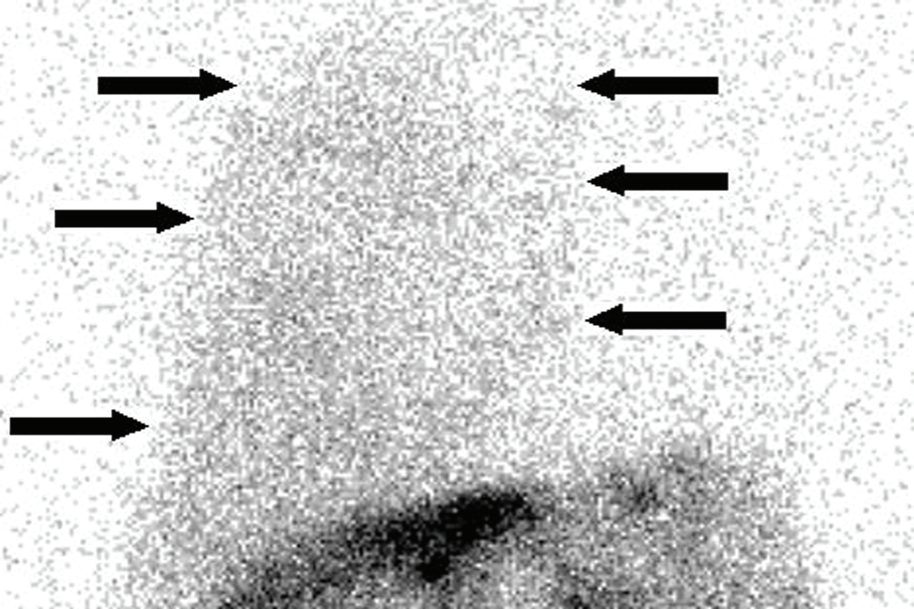
Peritoneal scintigraphy of the case 1 at 3 h: at 10 min, the tracer was diffused in the abdominal region, which confirmed that it was injected properly in the peritoneal cavity; at 3 h, the tracer was seen in the right side of the thoracic region (i.e. pleural space) (arrows). This image suggested peritoneo-pleural communication and hepatic hydrothorax.

**Figure 3:**
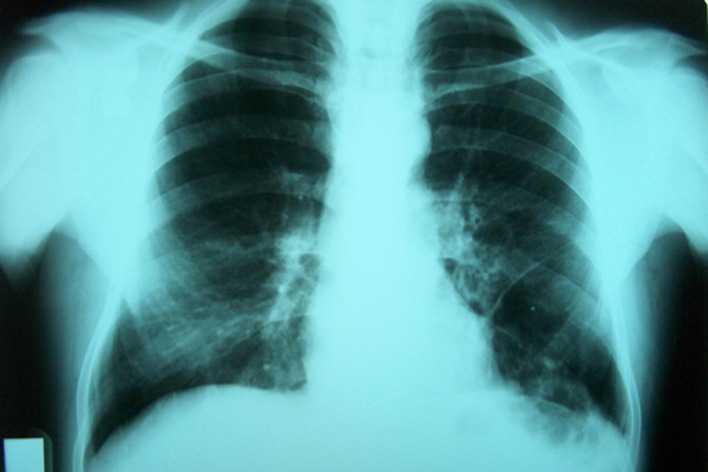
Post-treatment PA chest radiograph of the case 1: no evidence of pleural effusion was seen.

A 44-year-old male patient with a diagnosis of hepatitis B virus related cirrhosis of liver for 2 years was hospitalized with dispnea due to suddenly developed ascites and right sided pleural effusion. Posteroanterior (PA) chest radiograph of the patient demonstrated a large pleural effusion evident in the right hemithorax (Fig. 1). Prompt aspiration of thoracic fluid with paracentesis was performed. The biochemical examination of fluids revealed a transudate type pleural effusion with a high albumin gradient ascites (Table 1). After injection of ^99m^Tc sulfur colloid into the peritoneal space, rapid accumulation of radioactivity in the right pleural effusion was noted (Fig. 2), suggesting a peritoneo-pleural communication. With the treatment of ascites via paracentesis, salt restriction and diuretics ascites disappeared as well as the pleural effusion decreased to minimal levels. PA chest radiograph demonstrated the absence of the effusion (Fig. 3).

**Table 1:** Laboratory results of patients

	Case 1	Case 2
Age	44	53
Sex	Male	Male
Side of effusion	Right	Left
WBC	15300	3800
Hb	15.7	12.9
ESR	35	90
Serum albumin	2.9	3.1
SAAG	1.5	0.8
SPAG	1.1	0.4
INR	1.7	1.1
Leukocytes in ascites	-	507
Lymphocytes in ascites	-	190
ADA level in ascites	10	165

WBC: White blood cell; Hb: Hemoglobin; ESR: Eryhtrocyte sedimentation rate; SAAG: Serum ascites albumin gradient; SPAG: Serum pleural effusion albumin gradient; INR: International normalized ratio; ADA: Adenosine deaminase activity.

## Case 2

**Figure 4:**
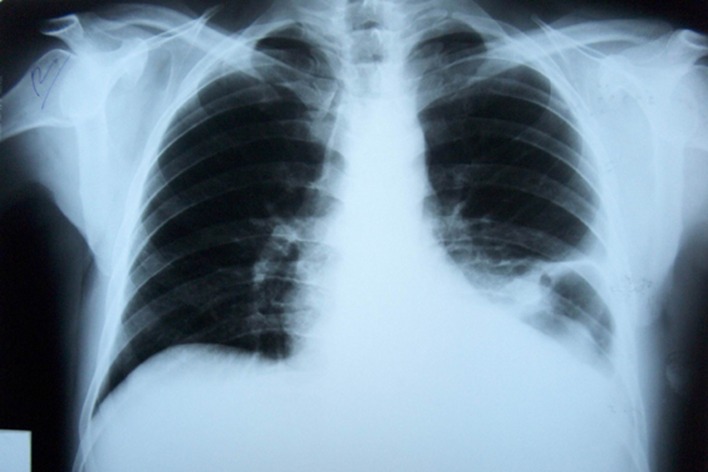
Pre-treatment PA chest radiograph of the case 2: a large pleural effusion evident in the left hemithorax was seen.

**Figure 5:**
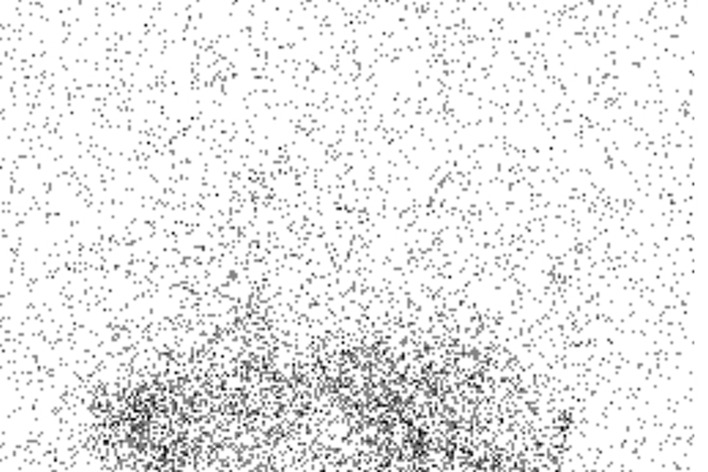
Peritoneal scintigraphy of the case 2 at 24 h: at 10 min, the tracer was diffused in the abdominal region, which confirmed that it was injected in the peritoneal cavity; at 6 h and 24 h, the tracer was seen in the region of abdominal cavity, but not in the thoracic region. This image suggested no peritoneo-pleural communication in the case 2.

**Figure 6:**
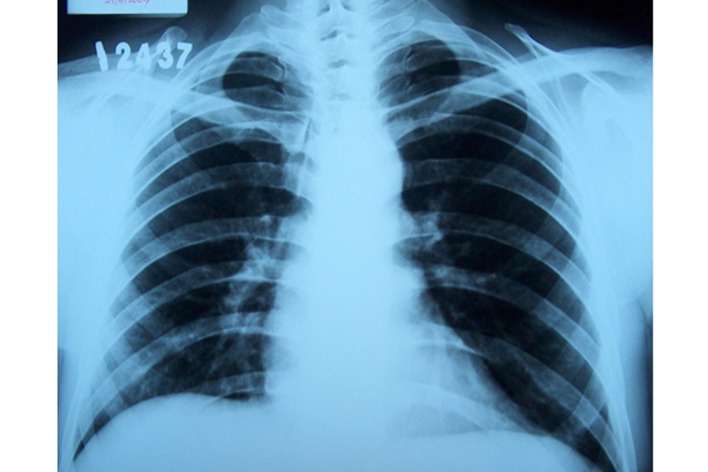
Post-treatment PA chest radiograph of the case 2: no evidence of pleural effusion was seen.

A 53-year-old male patient with newly developed abdominal distension, ascites and left sided pleural effusion was referred. During the diagnostic work up, he was found to have alcoholic liver disease. PA chest radiograph of the patient showed, clearly, a large pleural effusion in the left hemithorax (Fig. 4). Prompt aspiration of fluids revealed an exudate type pleural effusion with a low albumin gradient ascites (Table 1). After injection of ^99m^Tc sulfur colloid into the peritoneal space, no accumulation of radioactivity in lung region was observed up to 24 h (Fig. 5). According to the peritoneal scintigraphy, absence of peritoneo-pleural communication was considered and further diagnostic work up was initiated. Computed tomographic examination of thoracic and abdominal cavities did not yield certain diagnosis even though there was a fluid accumulation with fibrotic changes in pleural and peritoneal membranes. Broncoscopic evaluation of airways with aspiration of broncoalveolar fluid confirmed lung tuberculosis, and laparascopic evaluation of intra abdominal cavity with peritoneal biopsy confirmed peritoneal tuberculosis with final demonstration of tuberculosis bacilli itself. An anti-tuberculosis treatment was started. Post-treatment PA chest radiograph demonstrated the absence of the effusion (Fig. 6).

## Discussion

It is well known that ascites frequently forms in the setting of cirrhosis as a result of portal hypertension. Hypoalbuminemia, increased pressure in thoracic duct and azygos veins are common findings in cirrhotic patients with ascites. Furthermore, ascites can be complicated by high-output or low-output heart failure, nephrotic syndrome, malignancy, tuberculosis and some other infections such as chlamydia or coccidioidomycosis. Pancreatic or biliary ascites forms by leakage of pancreatic juice or bile into the peritoneal cavity or by a ‘chemical burn’ of the peritoneum. After abdominal surgery, especially extensive retroperitoneal dissection, lymphatics may be transected and may leak lymph for variable amounts of time [12, 13].

In daily clinical practice, pleural effusions with ascites are not uncommonly seen. Although mainly seen secondary to alcohol-related cirrhosis of liver, they can also be seen in viral hepatitis related cirrhosis [14]. They are usually unilateral and right sided but occasionally may be bilateral or left sided. A unilateral right sided pleural effusion with ascites is usually thought to be due to passage of ascitic fluid from the abdomen to the pleural space through acquired transdiaphragmatic defects [3]. Only the demonstration of these defects may clinically provide sufficient diagnostic criteria for hepatic hydrothorax, especially when there is no other clinical evidence away from liver disease. However, a unilateral left sided pleural effusion is usually poorly associated with these transdiaphragmatic defects and frequently needs further serious investigations.

The transdiaphragmatic defects probably result from anatomic thinning and separation of the collagenous fibers of the tendinous portion of the diaphragm [4]. Congenital factors, high intra abdominal pressure and prolonged bed rest may contribute to the diaphragmatic thinning [15]. Another possible explanation for the formation of transdiaphragmatic holes that allow the peritoneum to rupture into pleural space may be an increase of pressure caused by Valsalva maneuvers (cough, defecation, parturition) and trauma [16]. These defects are rarer on the left side because the left diaphragm is thicker and more muscular [17].

In clinical evaluation, practice of ordering every conceivable body fluid test on every pleural fluid specimen is expensive and seems more confusing than helpful, especially when unexpectedly abnormal results, such as left sided pleural effusions in cirrhotic patients, are encountered. A rapid and simple model method for establishing whether there is any communication between two cavities is to inject a radioactive tracer into the peritoneal cavity. Other diagnostic modalities of transdiaphragmatic fenestration have included intraperitoneal instillation of air or contrast agents, MRI and surgical exploration by thoracoscopy [17-19]. In order to prove peritoneo-pleural communication by peritoneal scintigraphy, several radiopharmaceuticals such as ^99m^Tc sulfur colloid, ^99m^Tc macroaggragated albumin (MAA) and ^99m^Tc albumin colloid have been used [9, 20]. We used ^99m^Tc sulfur colloid because of the ready availability and low cost. This technique is very easy to perform. It is safe, cheap, minimally invasive, and it has low radiation dose to the patient. For its effectiveness and meaningful results, peritoneal scintigraphy has already been used to diagnose of hydrothorax not only in patients with cirrhosis but also in patients on continuous ambulatory peritoneal dialysis (CAPD) [21].

Initial demonstration of the presence of this communication may play a vital role in patient management because it may not only stop further efforts to investigate other causes but also quickly start treatment. In our first patient, the pleural effusion was shown to be due to the passage of ascitic fluid across the diaphragm via the diaphragmatic defects or the lymphatic channels. In addition to clinical features and biochemical findings of ascites and pleural fluid, with the scintigraphic demonstration of transdiaphragmatic passage, patient was confirmed to have hepatic hydrothorax. In the second patient, there was not any communication seen between ascitic and pleural fluids. Lack of communication between two fluids led to separate investigations of thorax and abdomen. Invasive procedures, such as broncoscopy and laparascopic peritoneal biopsy procedures, were performed to further explore the patient. Only after the laparoscopic demonstration of tuberculous granulomas, certain diagnosis was established and anti-tuberculosis treatment was started.

In conclusion, use of ^99m^Tc sulfur colloid peritoneal scintigraphy to identify abnormal transdiaphragmatic communication between thoracic and abdominal cavities may play a critical role in the management of ascites with pleural effusion; it may help to start treatment quickly and, furthermore, may save healthcare resources.
